# No matter what the name, we’re all the same? Examining ethnic online discrimination in ridesharing marketplaces

**DOI:** 10.1007/s12525-021-00505-z

**Published:** 2022-01-26

**Authors:** Olga Abramova

**Affiliations:** University of Potsdam, Potsdam, Germany

**Keywords:** Sharing economy, Discrimination, Racism, Discrete choice experiment, Stated preferences, Social inclusion, M1, J15

## Abstract

Sharing marketplaces emerged as the new Holy Grail of value creation by enabling exchanges between strangers. Identity reveal, encouraged by platforms, cuts both ways: While inducing pre-transaction confidence, it is suspected of backfiring on the information senders with its discriminative potential. This study employs a discrete choice experiment to explore the role of names as signifiers of discriminative peculiarities and the importance of accompanying cues in peer choices of a ridesharing offer. We quantify users’ preferences for quality signals in monetary terms and evidence comparative disadvantage of Middle Eastern descent male names for drivers and co-travelers. It translates into a lower willingness to accept and pay for an offer. Market simulations confirm the robustness of the findings. Further, we discover that females are choosier and include more signifiers of involuntary personal attributes in their decision-making. Price discounts and positive information only partly compensate for the initial disadvantage, and identity concealment is perceived negatively.

“*In the long run, I certainly hope information is the cure for fanaticism,  but I am afraid information is more the cause than the cure.*”

*Daniel Dennett, an American philosopher, writer, and cognitive scientist*

## Introduction

Sharing marketplaces that connect individuals who possess idle resources with individuals who need those (Li et al., 2015) have refashioned consumption habits across a broad range of goods and services. Although human society has always been about joint effort (Belk, 2010), the advent of the Internet has introduced opportunities for that collaboration to happen at a greater scale and with more excellent connectivity. 72% of American (Pew Research Center, 2016) and more than 60% of consumers worldwide have successfully incorporated peer-to-peer sharing of apartments and rooms (e.g., Airbnb and 9flats), free car seats (BlaBlaCar), parking places (ParkatmyHouse), household devices and appliances (Zilok), and clothes (GirlMeetsDress) into their lives. Not by chance, the generated value of sharing platforms is projected to grow from $15 billion in 2014 to $335 billion in 2025 (PWC, 2015).

However, while the idea of sharing idle capacity has indisputable advantages, this concept is not without its challenges. Online platforms still face a barrier of information asymmetries, rooted in the fact that geographically separated agents make their choices under uncertainty (Dimoka et al., 2012). Indeed, when deciding whether to stay or drive with a stranger, both suppliers and applicants may feel ambiguity about another party, asset, and the overall experience of joint consumption. Hence, to tackle adverse consequences of uncertainty and promote trust, sharing platforms encourage self-disclosure and offer users a plethora of cues (e.g., Airbnb, 2020; BlaBlaCar, 2020). Notably, offline ID verifications, links to social media accounts (Pavlou et al., 2007), verified photos and videos of the apartments and people (Hong & Pavlou, 2010; Tang & Lin, 2016), as well as online feedback systems featuring opinionated reviews, star ratings and peer references (Benlian & Hess, 2011; Yang et al., 2018) are all assumed to translate into insightful signals that can be harnessed to compare offerings.

Despite the optimistic evidence that users’ disclosures prompt desirable outcomes like increased intention to transact and willingness to pay for a sharing offer (Abramova et al., 2017; Ert et al., 2016; Fagerstrøm et al., 2017; Yang et al., 2018), providing personal information can be a double-edged sword. Conceivably, the positive effect of revealing one’s own identity is counterbalanced by its discriminative potential, i.e., less favorable treatment based on innate characteristics or preferences (National Research Council, 2004), increasingly addressed by researchers across a variety of contexts. So far, disadvantageous treatments have been registered on AirBnB (Cui et al., 2019; Edelman et al., 2017; Kakar et al., 2018), Craigslist (Doléac and Stein 2013), Uber (Ge et al., 2020), Prosper.com (Pope & Sydnor, 2011) and BlaBlaCar (Farajallah et al., 2019). Moreover, prejudices were revealed on both sides of the market, i.e., toward both suppliers and consumers of the shared resource. Other studies, however, spotted no significant discrimination effects driven by disability status (Dai & Brady, 2019) or gender (Mejia & Parker, 2021). Overall, so far, the results remain mixed whether users account at all for others’ identity when contemplating their behavioral decisions on sharing platforms. With this study, we aim to add evidence on discrimination in the context of ridesharing platforms.

Methodology-wise, discrimination in sharing economy was often investigated with field experiments (e.g., Ahuja & Lyons, 2019; see Table 1 for review) and field studies (e.g., Kas et al., 2019; Tjaden et al., 2018), primarily drawing an inference about discrimination based on the observed outcome (e.g., a discrepancy in prices, response rate, waiting time). While having a great advantage of high external validity because of the natural settings, field studies and experiments do not allow to control for exogenous variables that may confound the results. Recently, vignette studies (a.k.a. factorial surveys), where participants have to put themselves in the scenario (a.k.a. situation or vignette) and reveal their judgments on the offered questions (Atzmüller & Steiner, 2010) were applied to study gender-related pay gaps (Auspurg et al., 2017) and antifeminist communication norms (Beyer et al., 2020). The complex description of the scenario makes sensitive attributes less obvious to a respondent. Within this method stream, discrete choice experiments (DCEs) (a.k.a. stated choice experiments) ask respondents to make choices in a scenario and allow reconstruction of preferences on the analytical stage. DCEs share the benefits of vignette studies and focus on choices instead of attitudes, thus being closer to approximating actual behavior (Liebe & Beyer, 2020). For this reason, a DCE is appropriate to look into socially undesirable behavior, including discrimination (e.g., Liebe & Beyer, 2020), and is used in the current study.

As a remedy against discrimination, experts advise removing demographic identifiers like pictures and names, replacing them with ID numbers or user names (Edelman et al., 2017; Farajallah et al., 2019). The recent study by Mejia and Parker (2021) submits that on UBER, reduced operational transparency eliminates bias at the ride request stage but does not cure post-acceptance racial and LGBT biases. While such concealment suggestions give platform providers approximate guidance, flashbacking to the early time of the Internet era, they are ambivalent for other stakeholders since both supply and demand sides of a sharing transaction will be exposed to a “concealment—adoption” trade-off. Our paper aims to address this gap in the literature.

In particular, this study aims to understand better discrimination and the effects of identity concealment in the context of ridesharing platforms—such as BlaBlaCar, Fahrgemeinschaft.de, Pop-a-Ride, Wunder Mobility, and Traeguate—that have been disrupting traditional city-to-city transportation industries worldwide, and especially in Europe. These platforms match the rider to available drivers who are independent contractors. The price is suggested by a driver and can be negotiable, while the platform advises on the appropriate compensation based on distance, driving experience, membership on the platform, car model, day and time, as well as other circumstances (e.g., luggage, pets, kids, etc.). Against this background, we ask the following research questions: *Are co-sharers (drivers and co-travelers) with perceived out-group names chosen less often than those with presumed in-group names in ridesharing platforms in Europe?* (RQ1). *And if so, how big is the effect of the prejudices against other factors, such as reputation or the price of a ridesharing offer? (*RQ2). *Is name non-disclosure more beneficial than name disclosure on ridesharing platforms in Europe?* (RQ3). *And if so, for whom?* (RQ4).

To answer them, we conduct a discrete choice experiment in the ridesharing domain on a European sample. Five characteristics of an offer are varied: (1) driver’s identity via name, (2) co-traveler’s identity via name, (3) driving experience, (4) reviews, and (5) price. It has been controlled for gender and experience in using ridesharing platforms. Finally, we asked participants about their attitude to concealment of the real names.

Investigation of discrimination implies opposition of at least two groups—the one that discriminates and the one which is discriminated. While the United States suffers from the confrontation “Whites vs. African-Americans” (e.g., Cui et al., 2019; Edelman et al., 2017), Europe is long concerned with immigrants coming from Middle Eastern, majority-Muslim countries, such as Syria, Afghanistan, and Iraq. These three countries accounted for more than 53% of all 2015–2016 asylum applicants in the EU (Konle-Seidl, 2018; Wike et al., 2016). In light of periodically recurring migrant crises in European history, including the last refugee crisis 2015–2016*,* coincided with the attacks in Paris and Brussels (Wike et al., 2016), in the European Union, there exists a belief that immigration increases the likelihood of terrorism, crime and safety concerns (Meltzer et al., 2018). Past studies show that immigrants from the Middle East are viewed as unwilling to participate in the broader society and adopt the nation’s customs and way of life (Strabac, 2011; Strabac & Listhaug, 2008; von Sikorski et al., 2017). These negative attitudes may be reproduced in daily exchanges like sharing transactions where no hard standards of service quality exist, compared to formal businesses. In Germany, signs of discrimination in ridesharing markets have been evidenced by opposing German vs. Turkish names (Carol et al., 2019; Kauff et al., 2013; Liebe & Beyer, 2020) and German vs. Arab/ Turkish/Persian names (Tjaden et al., 2018). In our study design, we opted for testing European vs. Middle Eastern descent names due to the recent outflow of migrants to Western Europe.

We make three contributions to the literature. First, we add to the discrimination in electronic markets literature by demonstrating the ethnicity-based and gender-based differential treatments among European consumers towards drivers of presumably Middle Eastern origin as judged by the name descent. Male drivers with Middle Eastern descent names are particularly undesired, as expressed in lower intention to book a ride. For price premiums, we triangulate the findings with prior studies (e.g., Liebe & Beyer, 2020).

Second, we are first to test the discrimination potential of co-traveler’s characteristics, which constitute the peculiarity of sharing transactions contrary to seller-buyer exchanges. By demonstrating the significantly lower willingness to book a trip if a male co-traveler with a Middle Eastern descent name has already reserved a seat in a car, we extend the potential discrimination victims circle beyond the supplier’s role.

Besides, this work contributes managerially by informing that the attitude to names’ concealment is overwhelmingly negative, thus questioning the success of concealment/anonymization strategy (e.g., via replacement through ID numbers or nicknames) as discrimination remedy, proposed in previous investigations (e.g., Ahuja & Lyons, 2019; Edelman et al., 2017). Our analysis should serve as an impetus for providers and regulators to reflect on the social inclusion issue, considering the increased severity of ethnicity-based discrimination during the COVID-19 pandemic on BlaBlaCar in France reported by Ivaldi and Palikot (2020), and re-examine the remuneration scheme. A substantial share of consumers is price-sensitive, meaning that monetary incentives can motivate them to withstand an ethnicity-based bias; in turn, the price premiums that rigid peers would like to pay for their taste can be redistributed via a platform to the disadvantaged party.

## Theoretical background

### Understanding the concept of discrimination and its mechanisms

The original meaning of discrimination as “the act of distinguishing” (Merriam-Webster.com, 2021) is of neutral connotation. Later it transmitted a positive perception of superiority status, and from the nineteenth century until now, the term is most widely used in the negative sense (Merriam-Webster.com, 2021).

In this paper, we use the economic science definition and understand discrimination as a situation where members of one group are treated differently (in most cases, less favorably) than members of another group with identical productive characteristics (National Research Council, 2004). This differential consideration is based on the actual or perceived membership in a particular group or social category. Social psychologists hold a slightly different view and define discrimination as one of the people’s common biases against others outside of their own social group, tabulating prejudice (emotional bias), stereotypes (cognitive bias), and discrimination (behavioral bias). The three types of bias are related, but they each can occur separately from the others (Dovidio & Gaertner, 2010; Fiske, 1998). For instance, sometimes, individuals have an adverse emotional reaction to a social group (i.e., prejudice) without knowing even the most superficial reasons to dislike them (stereotypes). Throughout this work, we focus on discrimination as less favorable treatment.

Pioneered by Becker’s seminal work on employer prejudice, which shows that discrimination in the marketplace decreases the real income of both minority and majority groups (Becker, 1971), the economics of discrimination divides the existing models into two classes: (1) competitive models, focusing on individual utility-maximizing behavior which can contain discrimination, and (2) collective models, which explore how groups behave against each other (Autor, 2003). The majority of analysis and debates revolve around (1) competitive models, theorizing on taste-based and statistical discrimination, depending on the mechanisms underlying decision-making.

Taste-based (or animus-based) discrimination happens when agents hold a ‘taste for discrimination’ (Becker, 1971), meaning favor or disfavor for a particular group not based on adequately justified factors. For example, if a passenger refuses to go with an Afro-American taxi driver because they are Afro-American, or if a hotel manager hires only blue-eyed females as receptionists because of the eye color, are forms of taste-based discrimination. In contrast, in statistical discrimination (Arrow 1971; Phelps, 1972), differential treatment of two groups does not require a taste for discrimination but has a market-based rationale (Arrow, 1998). Imagine the groups do really differ in their average productivity. The reason might lie in educational quality or culture and is unobservable. In the long run, the employers’ experience will motivate them to use the characteristics they can observe, like race or gender, as a proxy for the non-observable, real cause of the productivity gap (Arrow, 1998). In other words, it occurs when individuals, who possess limited information about the actual quality (e.g., skills or goodwill) of another party, employ demographic categories (e.g., race, gender, age) as a substitute for quality-related attributes. For example, some Middle Eastern countries do not allow women to fly because of the supposed inability to manage the aircraft, so a male pilot would be required to operate a plane (Yanıkoğlu et al., 2020). Driving license education services are offered to people 16–18 y.o. because careless and inexperienced driving is expected by default.

The consequences of discrimination and their scale are determined by its type. Taste-based discriminating individuals cause lower levels of transaction activity, suboptimal allocation of resources, and must fund the cost of their distaste out of their own pockets. Meanwhile, statistical discrimination is perceived by economists as endorsing because it represents the optimal solution to a signal extraction problem. A rational, risk-averse, utility-maximizing individual or a profit-maximizing firm’should’ statistically discriminate, treating people with the same expected quality identically and groups with different expected quality differently. This strategy is not driven by animus, so additional information about the quality of the affected party would reduce future discrimination (Cui et al., 2017).

Aligned with the established topology, taste-based discrimination on sharing platforms can be expressed in preferences for the guests/co-travelers of a specific gender, marital status, race, or social class based on the host’s/driver’s personal likes or dislikes. Statistical discrimination is practiced when a potential guest/co-traveler lacks information on a host/driver and uses group characteristics (e.g., race, gender, age) to infer expectations about the potential collaborator and make a decision. Since there is no evidence that in Europe, drivers of different ethnicity indeed deliver various ridesharing quality, and the nature of sharing interaction is rather short-term, we assume that the observed discrepancies are taste-based.

### Discrimination in the online environment

In the online environment, discrimination occurs by denigrating or excluding individuals or groups using symbols, voice, video, images, text, and graphic representations (Tynes et al., 2014). Virtual forms of discrimination occur in social networking sites, forums, discussion boards, text messaging, web pages, online videos, music, and online games, with sharing platforms being no exception.

In the beginning, ICT-mediated communication raised hope for actively mitigating the number of unfair treatments by eliminating specific cues from the conversation, thus resulting in a more egalitarian, color- and gender-blind electronic global village (Ess, 2001; Negroponte, 1995). However, despite the absence of visual signifiers of discriminative attributes in early virtual environments, studies demonstrate that race takes on a linguistic form across a range of online communication settings among both adults (Glaser et al., 2002; Kang, 2000; Nakamura, 2002) and adolescents (Daniels, 2009; Tynes et al., 2004, 2014). The latter are at higher risk because children and adolescents generally have less critical thinking skills than adults. Therefore, hate messages in their demographic can quickly spur a snowball effect with devastating consequences for the affected party.

As the Internet advanced and the level of connectivity increased, more and more businesses moved to a platform model that emerged as the new Holy Grail of value creation by enabling exchanges between strangers (Gray, 2016). The first generation of online markets did away with racial and gender discrimination (Fisman & Luca, 2016). To “break the ice” and confer trust before an exchange (Wang et al., 2004), extensive online disclosure of identity, verifications, and peer review systems have been proposed. Indeed, why stop at collecting feedback when a lot of potentially valuable details could be extracted from buyers’ and sellers’ identities? The former, however, cuts both ways and is assumed to backfire on the senders of personal information with its discriminative potential. In sum, many of the social norms and ills that exist offline are often revived in online communities (Burkhalter, 1999). This is increasingly evident as pictures, videos, and graphic representations of the body become more common in cyberspace (Ayres et al., 2015; Doleac et al., 2013).

Following this logic, we assume discrimination in today’s electronic markets and sharing platforms in particular. Past studies support this proposition, witnessing discrimination on crowdfunding platforms based on gender (Gafni et al., 2021) and race (e.g., Younkin & Kuppuswamy, 2018) and on the United States local advertisement websites based on race (Doleac et al., 2013). In online peer-to-peer lending in China, women are disadvantaged compared to men (Chen et al., 2017). While eBay auctions evidence race differentials (Ayres et al., 2015; Nunley et al., 2011), Nunley et al., (2011) point out that price discrepancies emerge only for sellers with low eBay feedback scores leading to conclude statistical discrimination. In the Israeli local online market for used cars, Zussman (2013) found that Jewish car sellers discriminate against Arab buyers, which, as further analysis suggests, is motivated by ‘statistical’ rather than ‘taste’ considerations. The audit of the United States online housing market concluded that Caucasian agents saw significantly more housing-related ads, while predatory rent-to-own programs were observed much often by African American users. Property recommendations are also biased, with more expensive goods exhibited to women (Asplund et al., 2020). Surprisingly, the online mental health care market investigation delivered mixed evidence: in study 1, no racial or gender disparities were observed, while study 2 suggests a hierarchy of accessibility. More educated help-seekers are preferred over less educated ones, and among those less educated requesters, black help-seekers with a caseworker received significantly fewer positive responses (Kugelmass, 2018).

Along with the expected applicability of patterns common to online two-sided marketplaces, sharing economy platforms do not support the transfer of ownership from one party to another, unlike regular cyber businesses (e.g., eBay). Next, sharing focuses on joint consumption and implies a higher intensity of interaction between the parties throughout the time spent together (Mittendorf et al., 2019). Thirdly, the quality of shared services is mainly unregulated (Sundararajan, 2014), which, along with the fact that the majority of participants are non-professionals (Kwok & Xie,^2019^; Li et al., 2016), may fuel consumer uncertainty. Altogether, these unique contextual characteristics may amplify participants’ willingness to assess a transaction’s prospects from peer’s identities.

## Past studies on discrimination on sharing platforms

Prior research on sharing platforms reported numerous cases of discrimination, with most data coming from accommodation sharing (e.g., Ahuja & Lyons, 2019; Cui et al., 2019; Edelman et al., 2017) or ridesharing (e.g., Farajallah et al., 2019; Simonovits et al., 2018; Tjaden et al., 2018) settings. These studies differ in discrimination attributes and their operationalization, methodology, measured discriminatory outcomes, and recommendations to lessen the bias in the future. Table 1 summarizes empirical research along these several critical dimensions.

**Table 1:** Summary of the existing literature on discrimination on sharing platforms

Study	Context	Method	Basis			Finding	Recommendation
			Race/Ethnicity	Gender	Other		
Ahuja and Lyons (2019)	Airbnb	FE		✓	✓	Male SSR guests are less likely to be accepted than male OSR guests and female SSR guests	- Concealment - Instant bookings
Brown (2019)	Uber, Lyft, Taxis	FE	✓	✓	✓	Discrimination towards black taxi riders emerges in higher cancelation rates and longer waiting times	NA
Carol et al. (2019)	Ridesharing	FE	✓	✓		No differences in response time, but German passengers have higher acceptance and response order than Turkish passengers. Female passengers are more likely to be accepted than males	NA
Cui et al. (2019)	Airbnb	FE	✓			African Americans are less likely to be accepted. A review on the guests’ page can reduce discrimination	- Reputation systems - Data verification
Dai and Brady (2019)	Rover, Fiverr	E			✓	No significant impact of disabilities on credibility perception and employment preference	NA
Edelman et al. (2017)	Airbnb	FE	✓	✓		African American guests are less likely to be accepted. Discrimination seems to be very costly for hosts	- Concealment - Instant bookings
Farajallah et al. (2019)	BlaBlaCar	FS	✓	✓		Driver’s ethnic background is the strongest demographic predictor of demand and revenue	- Concealment - Instant bookings
Farmaki and Kladou (2020)	Airbnb	I	✓	✓	✓	Despite the anti-discrimination policy, hosts discriminate by either rejecting reservations and/or setting their property in such a way that certain individuals are excluded	- Host–Guest fit - Interaction guidelines - Bias training - Inclusive language
Ge et al. (2020)	UberX, Lyft, Flywheel	FE	✓	✓		African Americans experience longer waiting times and higher cancellation rates. Female passengers deal with longer, more expensive rides	- Concealment - Fixed fares - Regulations - Data analysis
Goel et al. (2020)	Airbnb	CS	✓	✓		An incentive mechanism helps to prevent bias and make a truthful judgment. Otherwise, biases can be corrected ex-post	- Incentive mechanism - Bias correction
Greenwood et al. (2020)	Ridesharing	VE		✓		No gender bias in ratings was revealed for drivers Upon a lower quality experience, female drivers are disproportionately penalized	- Maintain transparency - Debiasing intervention
Kas et al. (2019)	Bikesharing	FS	✓	✓	✓	Tenants from ethnic minorities receive fewer ratings. A rating system cannot reduce these initial inequalities	NA
Kauff et al	Ridesharing	S, E	✓			Pro-diversity beliefs reduce discrimination	- Introducing positive beliefs about ethnic diversity
Laouénan and Rathelot (2020)	Airbnb	FS	✓			Statistical discrimination would disappear if unobservable factors were uncovered	- Full transparency
Liebe and Beyer (2020)	Ridesharing	E	✓	✓	✓	Discrimination can be explained by xenophobic attitudes and the lack of personal contact with “foreigners.”	- Concealment - Diversity advertisement
Lutz and Newlands (2018)	Airbnb	FG, S				Freedom of choice is fundamental in sharing economy	- Penalties - Regulations
Mejia and Parker (2021)	Uber	FE	✓	✓	✓	African Americans and LGBT supporters have higher cancelation rates. No gender differences. Bias exists during times of non-peak demand	-Full transparency or -No transparency
Moody et al. (2019)	Uber, Lyft	FS	✓			Discriminatory attitudes towards race do not hamper the first-time use but are negatively related to frequency of use, satisfaction, and continuance usage of experienced users	NA
Rosenblat et al. (2017)	Uber	CS	✓			Consumer-based rating system is prone to taste-based factors	- Reputation systems - Concealment - Data analysis
Schor (2017)	Airbnb, RelayRides, TaskRabbit	I			✓	Educated, white-collar providers, engaging in manual labor, leaving people of low income less chance to prosper	NA
Simonovits et al. (2018)	Ridesharing	FE	✓	✓		Interaction effect of gender and ethnicity. Arabic male testers having the lowest approval rate	NA
Tjaden et al. (2018)	Ridesharing	FS	✓			Discrimination against drivers of a seemingly Arabic/Turkish/Persian descent	- Profile presentation

*CS* case study, *FE* field experiment, *FS* field study, *E* experiment, *VE* vignette experiment, *S* survey, *I* interview, *FG* focus group, *SSR* same-sex relationship, *OSR* opposite-sex relationship, *NA* not available

### Attributes and operationalization of discrimination

Differential treatments in sharing arrangements are reported to occur on the basis of race (Cui et al., 2019; Pahuja & Tan, 2017), which is frequently investigated in combination with gender (Edelman et al., 2017; Farajallah et al., 2019; Ge et al., 2020) or social class (Moody et al., 2019). Further, one work suspects discrimination based on sexual orientation and gender, reporting male homosexual couples as a group experiencing difficulties when looking for a place to stay (Ahuja & Lyons, 2019). Interestingly, the platform worker’s disabilities have no statistically significant impact on the perceived credibility and hiring decisions (Dai & Brady, 2019).

Our review suggests that most studies (> 60%) focus on race as a dichotomy between whites and blacks (e.g., Brown, 2019; Ge et al., 2020). Therefore, there is a need to further scrutinize the multifaceted racial landscape and potential interactions among groups. For example, Simonovits et al. (2018) compare four ethnic groups and find that Arabic men have distinctly lower success rates than Russian, Dutch, or Chinese co-travelers. Next, a field study by Farajallah et al. (2019) that contrasts French vs. Arabic names came to a similar conclusion. The current study complements these insights, providing evidence from Europe.

Measuring discrimination characteristics, most studies infer membership of a particular group by analyzing the name (Cui et al., 2019; Edelman et al., 2017; Ge et al., 2020) or photo (Ge et al., 2020). This approach is common in the Information Systems (IS) literature and corresponds to how ordinary users make their judgments on online platforms (Rhue & Clark, 2018). Indeed, unless someone explicitly specifies their ethnicity or gender in the self-description fields, name and photo remain the most prominent anchors for online peers. The only exception in our sample is the Ahuja and Lyons (2019) study, where applicants’ orientation in the request texts was explicitly specified. To operationalize discrimination attributes, prior researchers use distinctively racial/gendered names, manipulate skin tone in pictures, or use facial recognition software. One potential caveat to be mentioned is, of course, that visual features enable only rough differentiation with mutually exclusive categories so that skin color differences within a race and mixed-race cases are disregarded.

### Recommendations to combat discrimination

Experts advise on several measures to alleviate discrimination on sharing platforms, including concealment, detailed profile presentation, and instant bookings. The most popular and conspicuous recommendation is concealment or removal of identity–revealing markers (e.g., Edelman et al., 2017; Liebe & Beyer, 2020; Rosenblat et al., 2017). Instead of real pictures and names, switching to nicknames (usernames) or user ID numbers and generic profile images is suggested. One potential drawback of this approach is increased anonymity, which endangers trust—the basis and “invisible currency” of all sharing platforms (Teubner & Flath, 2015). Other experts (Cui et al., 2019), as such, disagree with the concealment policy and believe that greater disclosure will supply more relevant information, which is supposed to reduce users’ dependence on membership of a specific group as a cue.

Further, it is also proposed to focus more on reputation systems as an external quality signal of collaborating peers (Cui et al., 2019; Rosenblat et al., 2017). This should shift users’ attention from characteristics like gender, race, ethnicity, or political affiliation, incentivizing stereotypical thinking to more prudent cues that facilitate rational decision-making. Liebe and Beyer (2020) offer that discriminatory attitudes can be changed with the help of diverse advertising images and slogans.

Finally, technological solutions like instant bookings (Ahuja & Lyons, 2019; Edelman et al., 2017; Farajallah et al., 2019) are proposed to combat discrimination. With this, the choice is transferred from an individual to a computer-based system, which confirms the request if a potential applicant meets the pre-selected criteria. The leading sharing platforms (e.g., Airbnb and BlaBlaCar) have already implemented this feature to reduce the choice burden of tolerant users and decrease confirmation time.

The observed disparity in the recommendations seems to stem from difficulties in detecting a particular type of discrimination and the possible co-existence of both types (Fisman & Luca, 2016). Most studies describe the challenge as almost insurmountable and cautiously warning that it is unclear whether statistical or taste-based differences are spotted (e.g., Edelman et al., 2017). Simultaneously, depending on the discrimination motive, ways to tackle the problem may be radically different. If it is caused by incomplete information, then extensive disclosure will provide trustworthy cues that outperform reliance on gender or ethnicity as a signal. If the antipathy is taste-based, more information hardly solves the problem.

## Methodology

To determine the effects of users’ identity information, a discrete choice experiment was conducted. Our approach fits well with studying and isolating discrimination effects because it identifies each attribute’s independent influence on respondents’ choices. In this section, we (1) familiarize a reader with the discrete choice experiment approach, with examples from ridesharing context; (2) describe how the model was specified to fit the aim of the study; (3) present experimental design and flow; and (4) describe sampling and sample characteristics.

### The discrete choice experiment approach

Unlike conjoint techniques, which are entirely statistical, DCEs lean on a long-established choice behavior concept, namely random utility theory (RUT) (Manski, 1977). RUT revolves around a rational individual who aims to maximize utility via the own choices. Precisely, a person *i* allocates to each alternative *j* in the choice set $C$ a utility ${U}_{j}^{i}$. In the ridesharing context, it means that a customer on an online platform, which represents a pool of all posted offerings, chooses from several ridesharing offers (a.k.a. alternatives or products or profiles) that meet the departure –destination and time criteria. In consonance with the economic theory of value, products and services in a DCE are understood as a bundle of attributes, i.e., “characteristics that give rise to utility” (Lancaster, 1966, p. 163). For example, a ridesharing offer encompasses attributes like price, car comfort level, driver’s experience, co-travelers’ presence, pick-up, and hop-off point. Accordingly, the utility of a product is the total of the utilities of its attributes. Here, a utility is a latent construct in the decision-maker’s mind, which can never be fully captured by the investigators (Louviere et al., 2010). It encompasses two pieces: systematic utility (explainable part) and a random residual (unexplainable part). Thus, ${U}_{j}^{i}$ can be written as:

1 $$U_j^i=V_j^i+\varepsilon_j^i=\sum\nolimits_j\beta_jX_j^i+\sum\nolimits_p\gamma_pZ_p^i+\varepsilon_j^i\; \forall\;j\in C$$

Systematic utility $V_{j}^{i}$ reflects attractiveness a decision-maker *i* associates with alternative *j.* $V^{i}_{j}$  is a function of *j*’s features, a.k.a. attributes $X_j^i$  which concern the alternative itself (e.g., quality, charge) and the personal characteristics (e.g., experience, gender) $Z_{p}^{i}$ . For numerical convenience, $V_j^i$ is linear-additive, with coefficients *β*_*j*_ reflecting an attribute’s contribution to the selection of an alternative *j*. γ_p_ denotes coefficients for the potential impact of individual characteristics (Potoglou et al., 2013). Random residual $\varepsilon_j^i$reflects all unknown details that affect decisions. Random part yields stochasticity of utility, enabling to forecast not choices but merely the probability with which a consumer *i* is going to select an alternative *j* given the choice set *C*. It happens when the utility of an alternative *j* exceeds the utility of other feasible alternatives:

2 $$p^i(j/C)=\Pr\;\left[U_j^i>U_k^i\; \forall\;k\neq j,k\in C\right]=\Pr\;\left[V_j^i-V_k^i>\varepsilon_k^i-\varepsilon_j^i\; \forall\;k\neq j,k\in C\right]$$

Suppose ${\varepsilon }_{j}^{i}$ is an i.i.d variable, which is “equivalent to assuming that the unobserved attributes have the same variance for all options in each choice set and that these attributes are uncorrelated over all the options in each choice set” (Street & Burgess, 2007, p. 59). Then, choices are described with the multinomial logit model (MNL). In the MNL family, the probability of picking an alternative *j* is gauged via the conditional logit model and (2) can be paraphrased as:

3 $$p^i(j/C)=\frac{\exp(\theta V_j^i)}{\sum_{k\in C^{}}\exp(\theta V_k^i)}$$

where $\theta$ is a scale parameter and equals one for a single sample or non-repetitive experiment (McFadden, 1973). In a nutshell, the DCE approach assumes choices happen due to discrepancies in utilities among alternatives caused by differences in utility for each attribute.

Once choices are registered, $\beta$ parameters and the probability that an alternative *j* will be picked are assessed. Next, imagine the cost (price) is included in a vector of the product’s attributes. In this case, one can use estimates to gauge respondent’s willingness to pay for changes in the level of a given attribute, i.e., marginal willingness to pay (MWTP):

4 $$MWTP=-{\beta }_{price}^{-1}ln\left\{\frac{{\sum }_{j}\mathrm{exp}\left({V}_{j}^{1}\right)}{{\sum }_{j}\mathrm{exp}\left({V}_{j}^{0}\right)}\right\},$$

where ${V}_{j}^{0}$ is the marginal utility of the reference level and ${V}_{j}^{1}$ is the marginal utility of another level of the same attribute (Potoglou et al., 2013).

Finally, estimated model parameters can be fit into a simulator to convert raw data into the most probable choices for a pre-defined set of alternatives. These “what-if” games have the highest managerial value. An assumption on how utilities transform into choices has to be particularized. We stipulate the logit choice rule, under which the calculated utility values are mean realizations of a random process (Lilien et al., 2006). The rule establishes the share of cases that an alternative $j$ generates the maximum utility in the set of offered products $J$, and therefore will be selected:

5 $${p}_{ij}=\frac{{e}^{{u}_{ij}}}{{\sum }_{j}{e}^{{u}_{ij}}},$$

where ${u}_{ij}$ is the estimated utility of product $j$ to customer $i$.

The market share for an alternative $j$ is obtained by averaging ${p}_{ij}$ across decision-makers. Precisely, individuals for whom a product provided the highest utility, added together, and the result is divided by the total sample size:

6 $${m}_{j}=\frac{\sum_{i=1}^{I}{w}_{i}{p}_{ij}}{\sum_{j=1}^{J}\sum_{i=1}^{I}{w}_{i}{p}_{ij}},$$

where ${m}_{j}$ is a market share of an alternative $j$, $I$ is number of individuals, $J$ is the number of product alternatives presented to a person, ${w}_{i}$ is the relative buying volume of an individual $i$, with the mean amount across all individuals indexed to 1, and ${p}_{ij}$ is the probability of picking a product $j$ by a person $i$ on a single-time deal (Lilien et al., 2006).

### Model specification

Conducting a DCE involves three key stages: (1) model specification, (2) experimental design, and (3) questionnaire development (Johnson et al., 2013; Rose & Bliemer, 2008). In the model specification stage, the selection of attributes and levels was based on a pre-test with 49 workers on the Prolific platform (Palan & Schitter, 2018; Prolific.co, 2021). Upon accessing the pre-test, respondents saw a description of the ridesharing platform and a scenario about a weekend trip from London to Manchester (for the exact wording, see “Experimental Design and Questionnaire Creation”). On a scale from 1 = “not important at all” to 5 = “absolutely essential,” we asked: “How important is the following information to be available on the platform to you when selecting a ridesharing offer?” Participants believed that the driver’s verification of email and phone number is crucial (M = 4.33, SD = 0.92), followed by driving experience (M = 4.25, SD = 0.99), driver’s name (M = 3.57, SD = 1.47), preferences (M = 3.57, SD = 1.26) and photo (M = 3.55, SD = 1.39). Co-travelers’ attributes were rated as of average importance: co-travelers’ name (M = 3.06, SD = 1.46), picture (M = 3.06, SD = 1.46), and preferences (M = 3.33, SD = 1.23). The same is true for car attributes: car’s model and color (M = 3.06, SD = 1.41), car’s photo (M = 3.51, SD = 1.37), and car’s year of production (M = 2.88, SD = 1.15).

We focused on the fictional scenario of a ridesharing app named “Join&Joy.” Considering the focus of the current study and following findings on critical features from the pre-test, the following five attributes were included in the experiment: (1) driver’s name, (2) driver’s experience, (3) driver’s feedback from past trips, (4) price, and finally (5) co-traveler’s name. The driver’s email and phone number verification were always present because it is a prerequisite for registration on most sharing platforms (Airbnb, 2020; BlaBlaCar, 2020). Photos were deliberately not included, i.e., set as avatars, because of their complex influence on purchase decisions and possible correlation with the driver’s and co-travelers’ identity, which violates attributes’ independence assumption in the experiment. The levels for the attributes were chosen as follows (see Table 2).

**Table 2:** Attributes and levels as presented to the respondents

Attribute	Levels
Driver’s name	1. ID number 2. Username (nickname) 3. European descent female name 4. European descent male name 5. Middle Eastern descent female name 6. Middle Eastern descent male name
Co-traveller’s name	1. ID number 2. Nickname 3. European descent female name 4. European descent male name 5. Middle Eastern descent female name 6. Middle Eastern descent male name
Driving experience	1. Newbee—less than 1 year of driving experience 2. Intermediate – 1 + years of driving experience 3. Expert – 10 + years of driving experience
Review	1. No reviews 2. 1 positive review 3. 5 positive reviews
Price	1. £ 17 2. £ 22 3. £ 27

*Driver’s name* is the main focus of our analysis since it has a discriminatory potential. Levels were designed to answer our research questions. In line with the ongoing discussions about real names’ concealment and replacement with neutral alternatives, an ID number was set as the baseline level. We took randomly generated four-digit numbers to define exact values. Besides, the username as an additional level was tested since past research mentioned it as a possible solution to the discrimination problem (von Essen & Karlsson, 2019). The other levels varied in terms of gender (male vs. female) and ethnicity (European vs. Middle Eastern descent).

To minimize confounding effects, names’ selection was undertaken with great care. We have chosen the names, frequently used in the Middle Eastern countries of recent outflow of migrants to Western Europe, to contrast them with the names of European descent. First, we gathered the ten most popular male and female names from the three largest Western European countries (the United Kingdom, Germany, and France) (behindthename.com, 2019). Next, we combined the lists per gender and removed full duplicates and similar names (e.g., Leo and Léo [removed]; Emilia and Emily [removed]). This step resulted in 26 European descent male names and 25 European descent female names. Then, the ten most popular names in Middle Eastern countries (Turkey, Afghanistan, Syria, and Iran) (behindthename.com, 2019; www.StudentsOfTheWorld.info, 2019) were pooled together. Removal of duplicates and spelling variations (e.g., Mohammad and Mohammed [removed], Mustafa and Mostafa [removed] yielded a sample of 32 Middle Eastern male names and 34 Middle Eastern female names (Appendix A). This list was then employed in a pre-test at Prolific (Prolific.co, 2021) to verify whether the names fulfilled our selection criteria. Middle Eastern countries in our name sample are overwhelmingly Muslim (share of Muslim population comprises 99% in Turkey, 99.7% in Afghanistan, 99.4% in Iran (nationsonline.org, 2021), and 87% in Syria (Central Intelligence Agency, 2021). In particular, we asked: “What origin and gender does the owner of the name most probably belong to?” (1 = European Male; 2 = European Female; 3 = European Gender-neutral; 4 = Muslim Male; 5 = Muslim Female; 6 = Muslim Gender-neutral 7 = None of the offered groups). Based on this pre-test (Appendix A, Table A2), we opted for names most often sorted into a typical category with regard to origin and gender, controlling for cases in which items were put into other categories (e.g., perceived as gender-neutral or of universal origin). This created four pools of names used in the experiment: European descent male names [Arthur, Henry, Oliver, Jacob, William, Alan, Jack, George, Ben, Paul, Felix], European descent female names [Emma, Olivia, Isabella, Emilia, Sofia, Chloé, Alice, Rose, Marie, Hanna, Amelia], Middle Eastern descent male names [Mohammad, Yusuf, Ahmet, Mustafa, Samir, Yaser, Rashid, Ömer, Emir] and Middle Eastern descent female names [Samira, Aditya, Aischa, Zahra, Hala, Isla, Amena, Soraya]. To corroborate that the selected names belong to an intended origin group, we have run a post-hoc test with 101 independent raters (Appendix D), asking: “To what group do the following names most probably belong with regard to origin and gender?” (1 = European descent male; 2 = European descent female; 3 = European descent gender-neutral; 4 = Middle Eastern descent male; 5 = Middle Eastern descent female; 6 = Middle Eastern descent gender-neutral 7 = None of the offered groups).

For the level of usernames (nicknames), we took 20 randomly generated nicknames (bestrandoms.com, 2019). To ensure semantic neutrality, two researchers independently rated them concerning their polarity (positive-neutral-negative). As a result, eight unique usernames with a neutral sentiment were selected for the experiment: ichthyic, sojourn, qweqwebozo, zonkedkeown, panaryguin, lothario, xibgrng, and krstdfg.

*Driving experience* represents a quality-related attribute of a driver and is assumed to signal a positive expected experience. As an alternative cue that shifts attention from signifiers of discriminative peculiarities, its paramount importance is postulated by past studies in the ridesharing context (Farajallah et al., 2019; Tjaden et al., 2018). Our levels correspond to the classification commonly offered by sharing platforms. Thus, we distinguish between a (1) “newbee” with less than one year of driving experience, (2) “intermediate” with more than one year of driving experience, and (3) “experts” who drive 10 + years.

*Driver’s feedback from past trips* is another quality-related attribute typically used on sharing platforms, whose importance has been consistently demonstrated by earlier research (e.g., Abramova et al., 2017; Chen & Chang, 2018; Ter Huurne et al., 2017). Moreover, many experts place their hopes in reviews as a remedy to lessen discrimination (see Table 1). For the experiment, the following values have been selected: no reviews, one positive review, and five positive reviews.

*Price* Following the recommendation of the largest ridesharing platform in Europe (BlaBlaCar, 2020), it was advised to charge £ 22 for the selected route to keep the offer financially attractive. According to the pre-test, the mean price that represents a good value for this route is £ 22.18 (SD = 7.20, median = 20); a price that is expensive, yet still acceptable is £ 31.86 (SD = 10.12, median = 30); an average price that is too cheap, thus raising doubts about quality is £ 9.86 (SD = 4.41, median = 10). Hence, we decided to provide price levels of £ 17 (platform recommendation minus £ 5), £ 22 (platform recommendation), and £ 27 (platform recommendation plus £ 5), which are also aligned with the pre-test values.

*Co-traveler’s name* is another attribute with discrimination potential. Although similar to the driver’s name, co-travelers presence reflects the singularity of sharing settings in contrast to seller-buyer exchanges. This information is typical for ridesharing services where people are supposed to travel with strangers. We used the same levels and the same pool of names as for the driver to manipulate this feature.

All in all, we believe the selected features create a balanced trade-off between the collaborators’ identity (of a driver and co-travelers) as the attributes of interest and other characteristics (Krasnova et al., 2014; Mihale-Wilson et al., 2017; Rose & Bliemer, 2008). Table 2 gives an overview of attributes and levels as presented to respondents.

## Experimental design and questionnaire creation

Upon accessing the survey, respondents were presented with a detailed description of the ridesharing platform, its functionality, and its value proposition. To avoid reputation effects likely for well-established platforms, we named our marketplace “Join&Joy.” The “look and feel” of the app and its functionality were kept similar to existing market players. Being guided by the appearance and availability of cues on real platforms, we included information about the trip, the driver, the co-travelers, and a booking opportunity. Next, the attributes and their corresponding levels were presented. We forced respondents to spend at least one minute on that page by hiding the “Next” button for 60 seconds.

All scenarios began with a setup in which participants were asked to imagine that they were planning a trip from London to Manchester and looking for a ridesharing opportunity as a cheaper way to travel. “*Now imagine the following situation: you are planning a trip from London to Manchester, UK. The distance between cities is 200 miles, which yields around 4 hours 40 minutes estimated travel time. Since you do not want to spend too much money, you decided to look for offers on "Join&Joy." As described on the previous pages, offers on "Join&Joy" imply traveling together with other people (the driver and occasionally with some other passengers)*.” Respondents expressed their opinion on the realism of this hypothetical situation. After that, they were asked to make 12 choices presented in a random order for each participant. Because of the impracticability of the full factorial design (i.e., 6 × 6 × 3 × 3 × 3 = 972 profiles in our study), the number of choice sets was derived via the D-efficient design, i.e., sufficiently low D-error. D-error is the most widely used efficiency measure, where alternative designs are compared based on the determinant of the asymptotic variance–covariance matrix (AVC) matrix (Rose & Bliemer, 2008).

Efficient designs for our experiment (2 attributes with 6 levels and 3 attributes with 3 levels) could be made with either 36 or 72 different profiles (a.k.a. alternatives) (SAS, 2019). A fractional factorial design was employed, and with 3 possible app profiles (a.k.a. alternatives) for each choice situation, 12 choice sets were produced. Specifically, in each choice set, respondents were asked to choose one traveling opportunity (“Which option do you prefer?”) with possible answers A, B, or C, and a “no choice” option (“None of them”) to cover situations where none of the presented offers was acceptable for a respondent. After the main part, participants were exposed to a manipulation check, where they had to sort names and usernames from the experiment into respective categories (“To what group do the following names most probably belong with regard to origin and gender?”, scale: European Male—European Female—Nickname/Pseudonym—Muslim/Arabic Male—Muslim/Arabic Female—None of the offered groups). In the end, we asked several questions about demographics, a propensity to trust, attitude to concealment of real names with ID numbers and nicknames, and participants’ opinions on which group experiences discrimination nowadays. The flow of the experiment is summarized in Appendix B.

## Sampling and sample characteristics

An online questionnaire was distributed via the Prolific platform (Palan & Schitter, 2018) in November 2019. During recruitment, we advertised the study as a poll about sharing platforms to avoid selection bias. Besides, workers who participated in the pre-test were excluded. Four pre-selection criteria were applied to define the audience: (1) participant is a fluent English speaker, (2) approval rate on Prolific platform is at least 90%, (3) the number of previous submissions on Prolific platform is at least 20, (4) participant’s nationality is West European (the United Kingdom, France, Germany, Switzerland, Ireland, Austria, Belgium, and the Netherlands). Participation was compensated with £2.50. In total, 312 people completed the survey. The following sorting criteria were applied: (1) duration longer than 8 min [0 observations were excluded]; (2) passed attention/bot check (“What is 12–8?”) [4 observations were excluded]; (3) absence of straightlining, i.e., when a respondent repeatedly chooses the same answer option [2 observations were excluded]. After deleting unusable cases, a final net sample of 308 observations was obtained. We double-checked for ethnicity and excluded answers like “Black African”, “Turkey”, “Arab Emirates”, “Chinese”, “Pashtun” (respondent’s spelling preserved) [34 observations], to ensure the West European sample. Finally, responses from 9 individuals who were unable to differentiate between a real name and a pseudonym/nickname and the name’s origin and gender, giving less than 60% of correct answers, did not pass the manipulation check and therefore were eliminated.

In total, 265 responses were used in the final analysis. This number surpasses the minimum sample size 500*6/(3*12) = 83.3 for DCEs (Orme, 2010), with the threshold computed as:

7 $$N\ge 500\bullet \frac{{L}_{max}}{J\bullet S},$$

where $N$ is the sample size, ${L}_{max}$ is the largest number of levels across attributes, $J$ is the number of alternatives presented at once, and $S$ is the number of choices (cards). The average duration of completing the survey was about 19 min (mean = 19 min 28 s; median = 17 min 35 s). 58.1% of our sample were female, and 40.8% were male, 1.1% belonged to other genders. In terms of age, 70.6% (n = 187) were between 18 and 40 years old (mean = 34.6, median = 33, SD = 12.1), which corresponds to the sharing services demographics, with a net median income £20,000–£34,999 per year (see Appendix C for more detail). The majority of the sample (54.7%, n = 145) had already tried ridesharing services, and 87 of 120 non-experienced participants could imagine using sharing platforms in the future. With attitudes towards the “Join&Joy” ridesharing app among respondents being favorable, the app’s average perceived usefulness reached 5.10 (SD = 1.17), assessed on a 7-point scale (Malhotra et al., 2005). The introduced scenario was estimated as pretty realistic (“It is realistic that I consider such a platform when planning this trip,” 1 = strongly disagree, 7 = strongly agree), with an average score of 4.58 (SD = 1.60).

## Results

### Model estimates and marginal willingness to pay

The data was analyzed using a conditional logit model as advocated by McFadden (1973) for its consistency with economic theory, more precisely, with a random utility framework (Hauber et al., 2016). The dependent variable reflects the probability of choosing a particular alternative, which for a rational individual relies on the disparity in utilities among alternatives. In our case, the utility function of a participant *i* choosing an app alternative *j* in a choice set *t* looks as follows:

8 $${U}^{i}_{{jt}}={{c}}_{j}+{\upbeta }_{1}{Price}+\upbeta { }_{2}{{Driver}}{^{\prime}}s{\;name}+{\upbeta }_{3}{{Cotraveler}}{^{\prime}}s{\;name}+{\upbeta }_{4}{Driving\;experience}+{\upbeta }_{5}{Reviews}+{\upvarepsilon }_{jt} ,$$

where ${\upvarepsilon }_{jt}$ is a random error term, which reflects the researcher’s inability to measure utility perfectly.

An overview of the results can be found in Table 3. Goodness-of-fit (GoF) measures evidence that the proposed model fits the data well: The adjusted Estrella index, which ranges from 0 (no fit) to 1 (perfect fit), reached 0.59, and McFadden’s statistic equals 0.28, which also satisfies the range [0.2–0.4] and therefore can be accepted as good (Louviere et al., 2010).

**Table 3:** Model estimates and marginal willingness to pay (MWTP) for the total sample and gender differences

Feature	Levels	Total (N = 265)		Females (N = 154)		Males (N = 108)	
		Estimate	MWTP	Estimate	MWTP	Estimate	MWTP
Driver’s name	ID number	Reference level		Reference level		Reference level	
	Nickname	− 0.09	£ − 0.62	− 0.04	£ − 0.30	− 0.16	£ − 0.82
	**European descent female name**	0.66***	£ **4.32**	0.78***	£ 6.12	0.47**	£ 2.37
	**European descent male name**	0.25*	£ **1.64**	0.29*	£ 2.26	0.20	£ 0.99
	**Middle Eastern descent female name**	0.27**	£ **1.74**	0.40**	£ 3.14	0.05	£ 0.25
	Middle Eastern descent male name	− 0.18	£ − 1.18	− 0.16	£ − 1.24	− 0.21	£ − 1.07
Co-traveller’s name	ID number	Reference level		Reference level		Reference level	
	**Nickname**	− 0.22*	£ − **1.43**	− 0.15	£ − 1.21	− 0.28^†^	£ − 1.45
	**European descent female name**	0.30**	£ **1.94**	0.43**	**£ 3.41**	0.08	£ 0.38
	**European descent male name**	0.16^†^	£ **1.08**	0.27*	**£ 2.10**	0.03	£ 0.15
	**Middle Eastern descent female name**	0.28***	£ **1.86**	0.38***	**£ 3.03**	0.12	£ 0.60
	**Middle Eastern descent male name**	− 0.23*	£ − **1.53**	− 0.18	£ − 1.39	− 0.30^†^	£ − 1.55
Driving experience	Newbee—less than 1 year of driving experience	Reference level		Reference level		Reference level	
	**Intermediate—1 + of driving** **experience**	0.90***	£ **5.90**	0.96***	**£ 7.54**	0.83***	**£ 4.25**
	**Experienced—10 years of driving experience**	1.73***	£ **11.33**	1.81***	**£ 14.32**	1.62***	**£ 8.24**
Review	No reviews	Reference level		Reference level		Reference level	
	**1 positive review**	1.00***	£ **6.54**	1.04***	**£ 8.18**	0.90***	**£ 4.57**
	**5 positive reviews**	2.07***	£ **13.61**	2.06***	**£ 16.28**	2.07***	**£ 10.53**
Price		− 0.15***		− 0.13***		− 0.20***	
GOF	Adjusted Estrella	0.59		0.5504		0.655	
	McFadden’s LRI	0.2786		0.2567		0.3277	

Significant at *** < 0.001; ** < 0.01; * < 0.05, ^†^ < 0.1 level, not significant otherwise

We observe that the majority of coefficients are significant at the 0.05 level. In terms of the *driver’s* personality, respondents do not differentiate between an ID number and a nickname, perceiving both as highly anonymized self-presentation. In contrast, the use of a real name when it is European descent female name (β = 0.66, p < 0.0001), European descent male name (β = 0.25, p = 0.014) or Middle Eastern descent female name (β = 0.27, p = 0.008) significantly increases utility and the probability of booking as compared to the reference level (ID number). The Middle Eastern descent male name’s effect is not significantly different from the baseline (β =  − 0.18, p = 0.106). As for *co-travelers*, not all disclosure may be beneficial: Participants were less willing to travel if they saw a person with a Middle Eastern descent male name (β =  − 0.23, p = 0.031) had already booked a ride. The company of a woman or a man with a common European descent name was perceived favorably. As expected, respondents reacted positively to the *driving experience*, linking it to the trip’s higher safety. *Positive feedback* from past travels also yielded consumer confidence (β_1pos_rev_ = 1.00, p < 0.000), especially when multiple peers consistently expressed appreciation (β_5pos_rev_ = 2.07, p < 0.000).

After estimating the effect of various attribute levels on the user’s utility, we also computed the marginal willingness to pay (MWTP) for a change in an attribute level according to the following formula (Kjær, 2005; Ryan et al., 2008):

9 $$MWTP=\frac{{\beta }_{attribute}}{-{\beta }_{price}}.$$

We observe that compared to the anonymous driver, with only the ID number visible, consumers valued an opportunity to be driven by females with European descent names at £4.32. If a driver has a European descent male name or a Middle Eastern descent female name, our sample was ready to pay an extra £1.64 and £1.74, correspondingly. The presence of a Middle Eastern descent male name in the description did not generate a price premium (although MWTP = £ − 1.18, meaning the requirement for compensation, the coefficient was found insignificant, p = 0.106).

In contrast, a Middle Eastern descent male name in the co-travelers’ description was a significantly undesirable option. Participants were willing to spend £1.53 less on such a ride than an entirely uncertain but neutral option (i.e., ID number). Interestingly, the nickname camouflage was also perceived negatively here (MWTP = £ − 1.43). If a traveling peer had a European descent female name, respondents would go for a small premium of £1.94. The same is true for a Middle Eastern descent female name (MWTP = £ 1.86) and a European descent male name (MWTP = £1.08) in the co-travelers’ section.

Not surprisingly*,* consumers value the driving experience and peer reviews most. Compared to a novice, a mid-level experience is worth £5.90, while substantial expertise is awarded £11.33 extra, hinting at safety being prioritized among respondents. Finally, one positive review increases willingness to pay by £6.54, compared to a listing without feedback from past trips. If several people confirm a positive experience, then the ridesharing journey is valued £13.61 more.

## Additional analyses

### Gender differences

To test gender differences in our European sample, we run the model on the subsamples according to the self-reported respondent’s gender (Table 3). Females appear to be more sensitive to whom they are traveling with than males. A driver’s European descent female name (β = 0.78, p < 0.000), European descent male name (β = 0.29, p = 0.03), or Middle Eastern descent female name (β = 0.40, p = 0.002) significantly increases the chances to be chosen by women compared to offers where only driver’s ID number is given. In contrast, men are almost indifferent and only express a higher willingness to travel with a female driver holding European descent name (β = 0.47, p = 0.003) compared to a reference level. The discrepancies remain for co-travelers: a company of a person with a European female name (β = 0.43, p = 0.001), European male name (β = 0.27, p = 0.019), or Middle Eastern female name (β = 0.38, p < 0.000) is positively related to the choice probability of female customers. The coefficients for offers that include drivers with Middle Eastern male names (female respondents: β =  − 0.16, p = 0.277; male respondents: β =  − 0.21, p = 0.242) or and co-travelers with Middle Eastern male names (female respondents: β =  − 0.18, p = 0.207; male respondents: β = − 0.30, p = 0.086) are negative for both genders as compared to the reference level (ID number), although they do not surpass the standard significance threshold.

### The role of user experience

Next, we control for user experience (e.g., Moody et al., 2019), which was assessed with the question “How often have you used ridesharing platforms in the past (e.g., Blablacar, Mitfahrgelegenheit, Poparide or Flinc)?” on a scale 1 = Never, and I cannot imagine to use them; 2 = Never but I can imagine to use them in the future; 3 = Rarely; 4 = Occasionally; 5 = Sometimes; 6 = Frequently; 7 = Usually; 8 = Every time. Respondents who never actually used ridesharing (answers 1 and 2) were assigned to the group “Non-experienced.”

Table 4 suggests that non-experienced respondents rely on attributes like driver’s and co-traveler’s names slightly more than experienced users. For example, when an experienced user is statistically indifferent between driver’s ID and European descent male name (β = 0.22, p = 0.11), a non-experienced user would strictly prefer to go with a person owning a European descent male name (β = 0.31, p = 0.038). While a female co-traveler with a Middle Eastern descent name is appreciated by both groups (β_expr_ = 0.20, p = 0.04; β_non-expr_ = 0.37, p = 0.001), for non-experienced users co-traveler’s European descent female (β = 0.38, p = 0.01) and European descent male (β = 0.28, p = 0.03) name significantly increase the chance of choosing an offer compared to reference level while the effect for experienced users is insignificant.

**Table 4:** Model estimates and marginal willingness to pay (MWTP) for experienced vs. non-experienced group

Feature	Levels	Experienced users (N = 145)		Non-experienced (N = 120)	
		Estimate	MWTP	Estimate	MWTP
Driver’s name	ID number	Reference level		Reference level	
	Nickname	− 0.04	£ − 0.24	− 0.11	£ − 0.76
	**European descent female name**	0.52***	£ 3.25	0.80***	**£ 5.58**
	**European descent male name**	0.22	£ 1.35	0.31*	**£ 2.14**
	**Middle Eastern descent female name**	0.27*	**£ 1.67**	0.26^†^	£ 1.79
	Middle Eastern descent male name	− 0.10	£ − 0.61	− 0.23	£ − 1.57
Co-traveler’s name	ID number	Reference level		Reference level	
	**Nickname**	− 0.13	£ − 0.82	− 0.27^†^	£ − 1.89
	**European descent female name**	0.21	£ 1.31	0.38*	**£ 2.63**
	**European descent male name**	0.05	£ 0.34	0.28*	**£ 1.97**
	**Middle Eastern descent female name**	0.20*	**£ 1.24**	0.37***	**£ 2.55**
	**Middle Eastern descent male name**	− 0.28^†^	£ − 1.73	− 0.14	£ − 0.99
Driving experience	Newbee—less than 1 year of driving experience	Reference level		Reference level	
	**Intermediate—1 + of driving experience**	0.81***	£ 5.03	0.97***	**£ 6.76**
	**Experienced—10 years of driving experience**	1.58***	£ 9.83	1.83***	**£ 12.80**
Review	No reviews	Reference level		Reference level	
	**1 positive review**	0.90***	**£ 5.61**	1.01***	**£ 7.08**
	**5 positive reviews**	2.09***	**£ 13.02**	1.97***	**£ 13.75**
Price		0.16***		− 0.14***	
GOF	Adjusted Estrella	0.6414		0.5211	
	McFadden’s LRI	0.3156		0.241	

Significant at *** < 0.001; ** < 0.01; * < 0.05, ^†^ < 0.1 level, not significant otherwise

### Market simulations

To gain a deeper understanding of how consumers balance involuntary personal attributes (e.g., name) and standard quality characteristics of a ridesharing offer (e.g., reviews and price), we conducted a series of market simulations. The regression estimates (Table 3) were used as a starting point. In each simulation, we contrasted several ridesharing offers and deliberately varied two attributes to demonstrate how these variations affect the distribution of most probable choices among consumers. In other words, market shares are computed as defined in Eq. (6), i.e., a percentage of individuals for whom a ridesharing offer would provide the highest utility among all decision-makers.

**Fig. 1:**
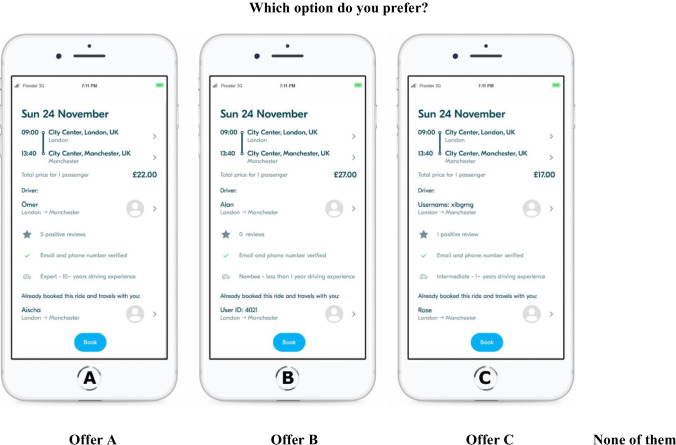
An example of a choice set presented to the participants

**Fig. 2:**
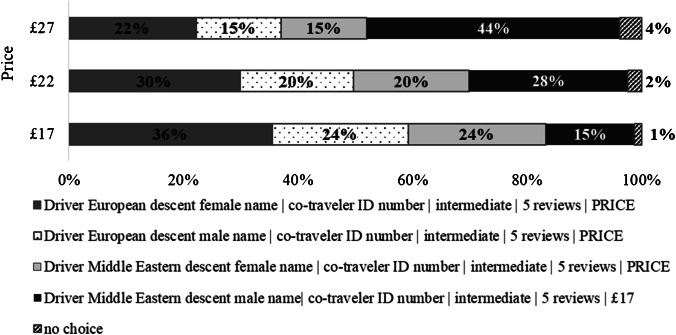
Market simulation 1: “driver’s name vs. price.”

In the first series of simulations, we aimed to investigate the effect of the driver’s name as a taste-based discrimination signifier on market shares. We simulated four ridesharing offers with different driver’s names: (1) driver with a European descent female name, (2) driver with a European descent male name, (3) driver with a Middle Eastern descent female name, and (4) driver with a Middle Eastern descent male name. In addition, it was accounted for the “no choice” option. For all offers, co-travelers were presented as ID numbers to minimize confounding effects, the level of expertise was set to “intermediate,” and the number of reviews was 5. The price for the fourth offer with a driver with a Middle Eastern descent male name is fixed at £17, while the prices of other offers varied. Figure 1 and 2 illustrates the four listings’ market shares (plus, a “no choice” option) as a function of price, shedding light on user behavior when confronted with the “driver’s name vs. price” trade-off.

We observe that if other attributes are set equal, the offer from a driver with a Middle Eastern descent male name will be chosen by 15% of consumers. A the same time, the offer from a driver with a European descent female name will most probably be selected by 36%, the offer from a driver with a European descent male name—by 24%, and the offer from a driver with a Middle Eastern descent female name—by 24% of consumers. As the price for a ridesharing opportunity from a driver with a European descent female name, a European descent male name, or a Middle Eastern female name increases, their market shares decrease. However, for 52% of consumers, a £10 price difference is not enough to compensate for aversion. If a male driver with a Middle Eastern descent name keeps the price at a relatively low level (here, £17) and other offers are available at £27, he can attract 44% of customers.

In the second series of simulations, subjective (i.e., driver’s name) vs. impartial (i.e., reviews) quality signals were explored. We again contrasted the ridesharing offers with four different driver’s names, keeping a “no choice” option. The price for all ridesharing options was set to £22 (the average level in our study); co-travelers were presented as ID numbers. The number of reviews was initially zero for all drivers and varied only for the male drivers with a Middle Eastern descent name.

**Fig. 3:**
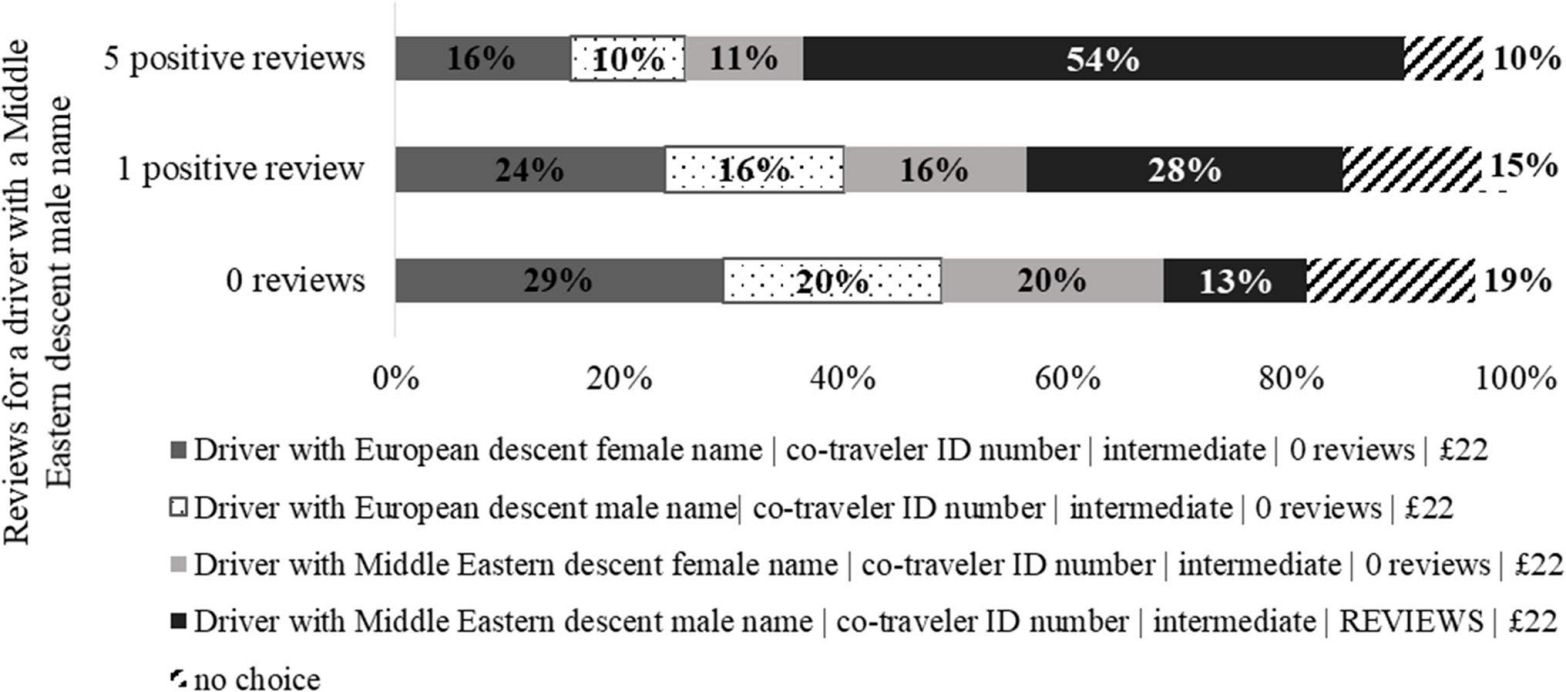
Market simulation 2: “driver’s name vs. reviews.”

As shown in Fig. 3, selecting among drivers without reviews from past trips, users are inclined to opt for a driver with a typical Middle Eastern descent male name less often (13%) as compared to drivers with a European descent female name (29%), a European descent male name (20%), or a Middle Eastern descent female name (20%). However, even one positive review turns the alignment of forces, giving the largest market share (28%) to an offer from a male driver with a Middle Eastern descent name. This indicates that some consumers rely on impartial cues if present. Positive feedback from past trips is an important reason to give up ethnic prejudices for 41% of consumers (54–13%), whereas 37% (16% + 10% + 11%) would nevertheless prefer drivers with European descent male names or Middle Eastern descent female names with zero reviews.

**Fig. 4:**
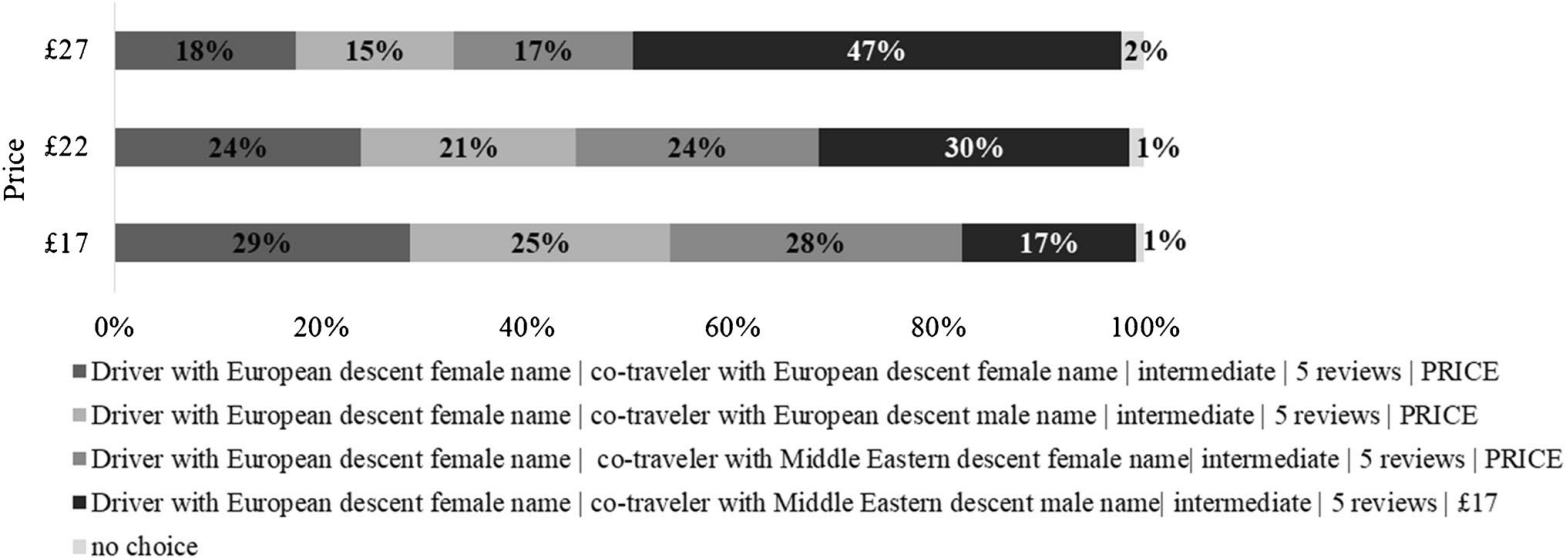
Market simulation 3: “co-traveler’s name vs. price.”

In the third series of simulations, the “co-traveler’s name vs. price” trade-off was addressed (Fig. 4). The ridesharing offers differed by a co-traveler’s name: (1) co-traveler with a European descent female name, (2) co-traveler with a European descent male name, (3) co-traveler with a Middle Eastern descent female name, and (4) co-traveler with a Middle Eastern descent male name. A “no choice” option was also considered. For all offers, drivers were set to have a European descent female name (most desired level in our sample), the level of expertise was set to “intermediate,” and the number of reviews was 5. The price for the offer with a co-traveler with a Middle Eastern descent male name (presumably treated less favorably, as regression results in Table 3 suggest) was fixed at £17, while the prices of other offers varied.

We observe that at a low price level (£17), the offer with a co-traveler with a Middle Eastern descent male name is least attractive, with a potential share of 17%. Other offers would be nearly twice more popular: 29% of consumers would book a trip with a co-traveler holding a European descent female name, 25% of consumers—with a co-traveler holding a European descent male name, and 28% of consumers—with a co-traveler having a Middle Eastern descent female name. When the prices of the first three opportunities increase to £22, and the offer with a co-traveler with a Middle Eastern descent male name is still available at £17, it slightly dominates the market with a share of 30%. The larger the price gap, the more customers would accept a ridesharing option with a co-traveler with a Middle Eastern descent male name for £17. If the price of the first three offers increases to £27, 47% of consumers would choose to go with a co-traveler with a Middle Eastern descent male name but at the lowest price (£17). Overall, simulation 1 and simulation 3 suggest some price sensitivity among consumers. However, price premiums tested (£5 and £10) are not enough to eliminate taste-based bias completely.

### Follow-up analysis on concealment as a possible remedy for discrimination

Follow-up analysis addresses the search for a plausible remedy for discrimination. The overview of past studies (Table 1) suggests concealment as a popular recommendation. Thus, respondents’ attitude to concealment was extracted with two questions: “What do you think of the idea to replace real names of users with ID numbers on sharing platforms?” and “What do you think of the idea to replace real names of users with nicknames/pseudonyms on sharing platforms?” on a 7-point scale (1 = Absolutely inappropriate; 2 = Inappropriate; 3 = Slightly inappropriate; 4 = Neutral; 5 = Slightly appropriate; 6 = Appropriate; 7 = Absolutely appropriate).

**Fig. 5:**
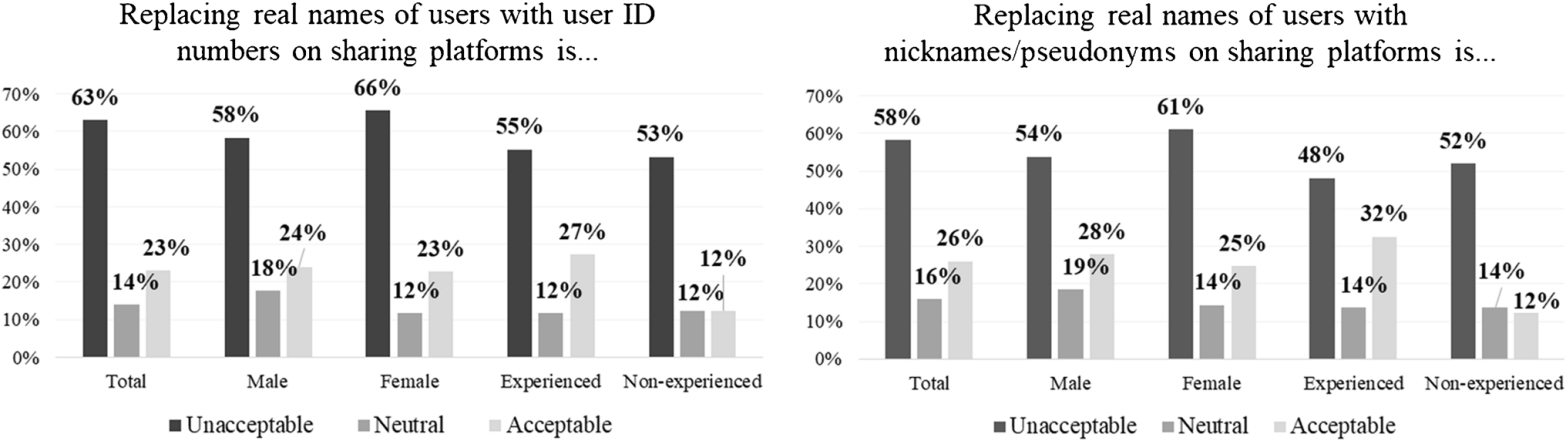
Attitude to concealment

We observe negative attitude to the replacement of real information with ID numbers or nicknames/pseudonyms. The replacement of real information with ID numbers is met negatively (mean = 3.29, SD = 1.66) regardless of gender (mean _female_ = 3.24, SD _female_ = 1.67; mean _male_ = 3.40, SD _male_ = 1.67) or experience (mean _expr_ = 3.52, SD_expr_ = 1.73; mean_non-expr_ = 3.03, SD_non-expr_ = 1.54). The same is true about replacement of real information with nicknames/pseudonyms (mean = 3.35, SD = 1.58), with gender (mean _female_ = 3.27, SD _female_ = 1.60; mean _male_ = 3.45, SD _male_ = 1.54) or experience (mean _expr_ = 3.64, SD_expr_ = 1.69; mean_non-expr_ = 3.00, SD_non-expr_ = 1.38) playing no role for the attitude. Figure 5 summarizes the distribution of attitude to concealment variables. For simplicity, answers 1 = Absolutely inappropriate; 2 = Inappropriate; 3 = Slightly inappropriate have been clustered as “Unacceptable” and answers 5 = Slightly appropriate; 6 = Appropriate; 7 = Absolutely appropriate as “Appropriate”.

## Discussion, implications, and concluding remarks

### Discussion of the study’s findings

We aimed to shed light on whether ethnicity- and gender-revealing cues of ridesharing platform participants, i.e., drivers and co-travelers, significantly affect peers’ choices expressed in a willingness to accept a ridesharing offer (RQ1), thus exhibiting the presence of discrimination in these markets, and how do they measure against other cues (RQ2). This study also sought to advance our understanding of the potential of the frequently proposed recommendation to handle discrimination, namely identity concealment (RQ3, RQ4). Due to the topicality of migrant crises in Europe, especially from Middle Eastern countries with the majority-Muslim population, we manipulate European vs. Middle Eastern descent names of drivers and co-travelers together with their gender. The results from a discrete choice experiment (DCE) raise several points of interest.

First, we observe a preference to go on a trip with a female driver with a common European name, followed by the one with a European descent male name and a Middle Eastern descent female name. Willingness to book a trip with a driver holding a Middle Eastern descent male name is not significantly higher than that of an anonymous agent. The finding may be interpreted as taste-based discrimination towards males of Middle Eastern origin, which is in line with Farajallah (2019), Liebe and Beyer (2020), Simonovits et al. (2018) and Tjaden et al. (2018), reporting disadvantages for male drivers from a seemingly Arabic/Turkish/Persian descent.

Second, when it comes to knowing who accompanies the trip, there is a preference to travel with people having European descent names regardless of gender or having a Middle Eastern descent female name but not with the owner of a Middle Eastern descent male name. Moreover, a significant aversion towards an offer with a co-traveler with a Middle Eastern descent male name is expressed compared to the case when a co-traveler’s identity is not disclosed. Derived from stated preferences, MWTP informs about demanded discounts to ride together with Middle Eastern male passengers. Thus, we observe that in ridesharing, European users are driven by homophily preferences (McPherson et al., 2001) and discriminate against people of a seemingly Middle Eastern origin who are perceived as an out-group presumably due to a different culture and religion, which, in turn, is projected to values and behavior. This evidence allows getting a yea to RQ1 concerning the presence of ethnicity-based discrimination.

Third, we observe that willingness to travel with females drivers is higher than with male drivers of the same origin. The same is true about co-travelers. For European ladies in our sample, although positively inclined towards the in-group in general, the positive effect of drivers with a European descent female name is stronger than that of drivers with a European descent male name. Consequently, MWTP for a driver with a European descent female name is about three times higher than for a driver with a European descent male name. For European male respondents, differences are significant only within the perceived European drivers: an offer from a driver with a European descent female name is significantly better than the one where the driver’s ID number is available. In contrast, European male disclosure is statistically indifferent from non-disclosure. In the out-group, the choices of European male respondents do not evidence preference towards a certain gender. This favors the explanation proposed by Liebe and Beyer (2020) that women might feel safer with other women as drivers. An alternative reason, “benevolent sexism,” cannot be fully accepted given the results. As an answer to RQ1, we observe the presence of gender-based differential treatment, with women being more favored as drivers and co-travelers.

Simultaneously, experienced users strongly prefer a female driver, which may imply that women are perceived as more accurate, punctual, and attractive drivers by this sub-group. For non-experienced respondents, the probability of booking a ride with a driver holding a European descent name is significantly higher. Drivers with Middle Eastern descent names, regardless of gender, do not increase the chances of an offer to be chosen compared to the default (driver’s ID number), hinting at aversion towards ethnicity.

As for the magnitude of effects (RQ2), we observe the impact of names (absolute β varies from 0.22 to 0.66) is significant, and is twice stronger than the impact of a £5 price difference, but is nearly 30% weaker than the impact of 1-year driving experience and 1 positive review. Moreover, examining female and male consumer choices reveals that females are choosier and include more signifiers of involuntary personal attributes in their decision-making than males do. Such a pattern may be explained by higher safety concerns women experience about transport (Women on the move, 2018).

Further, to elaborate on RQ2, we conducted a what-if analysis in the form of market simulations to test consumer sensitivity to signals like price and positive reviews. In the absence of taste-driven reasons (i.e., consumer’s indifference to driver’s origin), demand is highly price-elastic, meaning that a slight discount lures away nearly all customers from competitors. We observe that all other parameters set equal, a ride with a male driver holding a Middle Eastern descent name will be chosen in only 15% of cases, which is significantly behind offerings from drivers with names of another origin (European descent female name 36%; European descent male name 24%; Middle Eastern descent female name 24%). A £5 discount by a male driver with a Middle Eastern descent name doubles his prospective share (from 15 to 28%). If the gap becomes £10, 44% of consumers would prefer to go for a trip from a person with a Middle Eastern descent male name. However, 52% are sustainable in their aversion, would pay significantly more not to be driven by a male with a Middle Eastern descent name. “Antipathy” for male co-travelers with Middle Eastern descent names evidenced similar patterns and the range of values. Thus, taste-based discrimination is an issue in our sample, even keeping in mind inaccuracies due to imperfect human rationality or a hypothetical scenario. About one-half of respondents are insensitive even to the £10 discount.

As for the compensating effect of positive reviews, the findings are in the middle between two extremes from past research. Unlike Tjaden et al. (2018), who found that discrimination almost disappears if individuals have (more) positive information about the person, we do not observe the strong effects of positive reviews. At the same time, there is no full alignment with Kas et al. (2019), who report the reputation system’s complete inability to reduce ethnicity-based inequalities even with time. The second series of simulations suggest that 5 positive reviews of a male driver with a Middle Eastern descent name significantly increase the market share of the offer (from 13 to 54%) than offers without reviews. However, for 37%, this signal will not be strong enough. Therefore, a discriminatory disadvantage may only be partly eliminated with past positive feedback.

Concerning identity disclosure vs. concealment (RQ3, RQ4), our analysis points to ambivalence. On the one hand, real names are preferred by consumers compared to ID numbers or nicknames. Moreover, in the follow-up survey, the attitude to concealment is primarily negative. Therefore, our findings question the appropriateness of concealment strategy as a remedy against discrimination, though often proposed by previous studies (e.g., Ahuja & Lyons, 2019; Edelman et al., 2017). The experiment suggests replacing the actual name with a pseudonymous signifier (ID number or a nickname) can have positive or negative implications, depending on whose identity is hidden. The booking probability is higher if consumers see a European descent female name or a European descent male name, or a Middle Eastern descent female name. For drivers with a Middle Eastern descent male name, the effect of concealment is statistically identical to reveal, while drivers with common European descent names or females with Middle Eastern descent names will be worse off because of their anonymity. From the co-traveler’s perspective, non-disclosure of a male with a Middle Eastern descent name is beneficial if a real name is substituted with an ID number.

### Implications for theory

First, we add to research on discrimination in electronic markets and specifically on sharing economy platforms (Edelman et al., 2017; Mejia & Parker, 2021). Previous investigations focus on the African-American minority within the United States sample (Cui et al., 2019; Ge et al., 2016) and Arab/Turkish/Persian names in Germany (Carol et al., 2019; Kauff et al., 2013; Liebe & Beyer, 2020). Our paper complement the empirical evidence for revealing discrimination tendencies of West European (the United Kingdom, France, Germany, Switzerland, Ireland, Austria, Belgium, and the Netherlands) respondents, expressed in the lower willingness to book a ridesharing offer with peers holding typical names of Middle Eastern descent as compared to European descent names. Next, we triangulate the findings with other works. Since MWTP for ethnicity-related attributes in our sample varies from £ 1.64 (7.5% of the average price of £22) to £ 4.32 (19.6% of the average price of £22), the range of our values is more aligned with Liebe and Beyer (2020), who report a bonus of €0.48 (German vs. Italian drivers) and €1.26 (German vs. Turkish drivers), than with 32% discriminatory price premium obtained from market data in Tjaden et al. (2018).

Second, to the best of our knowledge, our investigation is the first to test the role of the discrimination potential of co-traveler’s attributes, which reflects the nature of sharing platforms. Prior designs perceived a sharing transaction as a driver–passenger interaction, disregarding co-travelers, who may substantially contribute to the overall ridesharing experience (Tjaden et al., 2018). We found that discrimination is not role-specific and, similar to drivers, male co-travelers with Middle Eastern descent names were associated with lower acceptance rates. This implies the importance of resolving uncertainty about co-sharers as a peculiarity of sharing arrangements. Although not addressed in previous works, this result points out that the boundaries of social sensitivity stretch beyond the supplier (i.e., a driver or a host) but include all participants involved in sharing.

Finally, on a broader scope, by examining the effects of identity cues on consumer choices in terms of acceptance and willingness to pay on sharing platforms, we reply to the call by Trauth (2017) for IS research on social inclusion/exclusion. Our “quantitative research can be useful in articulating *what is the problem* with respect to imbalance” (Trauth, 2017, p. 15). The finding on the negative attitude towards concealment contributes to the question of “what needs to be done to ameliorate this imbalance” (Trauth, 2017, p. 15) and stimulates further research.

### Implications for practice

The current study has practical implications. For users of ridesharing services, our results should raise awareness of the discrimination trends and motivate critical thinking rather than relying on heuristics in their future judgments. We do hope that consumers will ask themselves twice before booking: “Do I really have good reasons to reject a ridesharing offer observing a co-traveler or driver with a Middle Eastern descent name?” and inspect more objective predictors of quality like reviews and experience in driving or ridesharing.

Platform providers and policy-makers should be aware that, as of now, ridesharing space is not that inclusive as intended. Moreover, recent evidence from BlaBlaCar trips in France by Ivaldi and Palikot (2020) is alarming and suggests that the COVID-19 pandemic has reinforced ethnic discrimination. Further, our research informs platform providers and regulators that the attitude to names concealment is primarily negative. Therefore, the findings question the appropriateness of concealment/anonymization strategy (e.g., via replacement through ID numbers) as a remedy against discrimination, though often proposed by previous studies (e.g., Ahuja & Lyons, 2019; Edelman et al., 2017). An ID number is equally good as real names for male drivers of Middle Eastern descent but is significantly worse for drivers with European descent names of both genders and drivers with Middle Eastern descent female names. Male co-travelers with Middle Eastern descent names will be vice versa, better off hiding their own identity behind an ID number, while other groups will be worse off of this policy. This hints that unified regulation (“one size does not fit all” approach) can be problematic. In this vein, when designing platform rules, it is advisable to allow users to choose how to present themselves.

At the same time, we demonstrate that impartial cues like positive reviews and expertise (in our case, driving experience) can, to some extent, compensate for initial equalities. This should motivate socially responsible providers to visually design the platforms so that more attention is attracted to reviews and expertise, e.g., making names size relatively small. Finally, because monetary incentives can motivate some users to withstand an ethnicity-based bias, platform providers can adjust the pricing scheme. As for the rigid users, their price premiums for taste can be redistributed via a platform to the suffering party.

## Limitations and future research

This study has several limitations, which, however, offer exciting avenues for future research. First, our experiment was conducted for a ridesharing marketplace, raising possible concerns on the transferability of our insights to other contexts. For additional settings (e.g., accommodation-sharing) with different perceived risks and prices, results may vary. Therefore, a comparison between industries is worth consideration as well. Second, a simplification of a ridesharing offer presentation has been done to fit all data into one screen (see Fig. 1). In this regard, users were not provided with information about a car, which may still convince some consumers, although shown to be of marginal importance in the pre-test. Next, the participants were recruited from a single participant pool of Prolific. Although we took precautions to ensure data quality, further research is needed to show if our results hold for other samples.

Further research may account for factors like distance and time spent together during sharing (e.g., long vs. short trips and long vs. short stays) and similarity/homophily effects (e.g., based on ethnicity or gender). The investigation of the opposite side, i.e., checking whether Middle Eastern descent participants discriminate against Europeans, would be of particular interest (Liebe & Beyer, 2020). This could shed light on the mutuality of discrimination, whether aversion is relative and holds for an out-group or absolute for a particular group. To disentangle statistical and taste-based bias, a mixed-method approach combining big data analysis of existing offers and experimental design (e.g., Younkin & Kuppuswamy, 2018) can be appropriate. Finally, while we found aversion towards the names’ concealment, the efficacy of other designs and platform policy choices against discrimination outcomes (e.g., see a taxonomy by Levy & Barocas, 2017) needs empirical assessment.

## Conclusion

Sharing platforms bring strangers together to temporally enjoy the benefits of collaboration and reduce average costs. Online identity disclosure builds ex-ante trust but can backfire with its discriminative potential. In ridesharing, participants with European descent names are reluctant to male peers with Middle Eastern descent names, and the nature of this discrimination is taste-based. Price discounts and positive information about the person, like positive reviews and extensive driving experience, only partly counterbalance the initial disadvantage but do not let it disappear entirely. Concealing real names with ID numbers, nicknames, or pseudonyms, which equalized everyone, is met negatively. In a social marketplace like sharing platforms, effort should be put to withstand an ethnicity-based bias to achieve sustainability.

## Appendices

## Figures and Tables

**Fig. 6:**
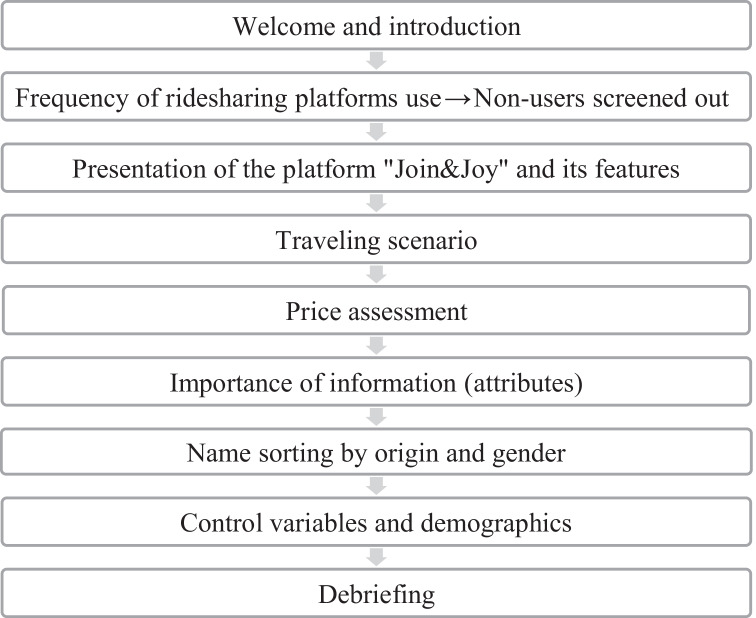
Flow chart of the pre-test

**Fig. 7:**
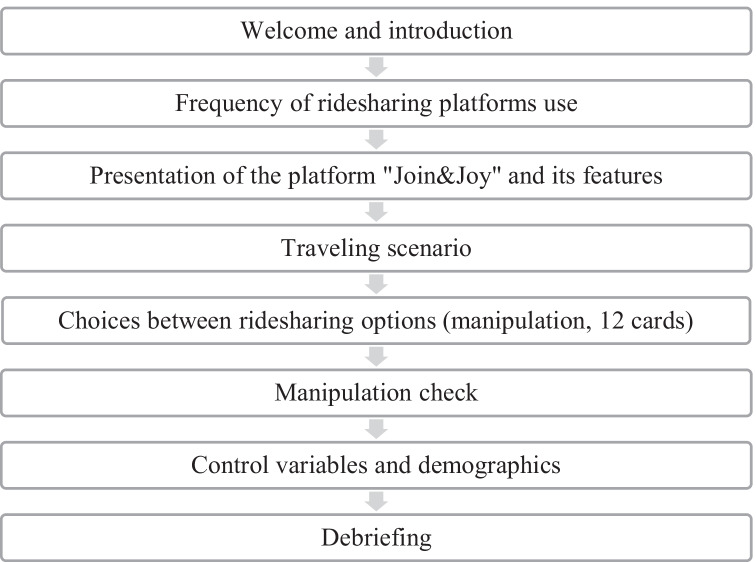
Flow chart of the experimental procedure

## Appendix A: Overview of the pre-test

See Fig. 6 and Table

**Table 5:** Survey instrument for the pre-test

**Frequency of ridesharing platforms use** How often have you used ridesharing platforms in the past (e.g., Blablacar, Mitfahrgelegenheit, Poparide or Flinc)? (1 = Never and I cannot imagine to use them; 2 = Never but I can imagine to use them in the future; 3 = Rarely; 4 = Occasionally; 5 = Sometimes; 6 = Frequently; 7 = Usually; 8 = Every time)
**Price assessment** For the scenario mentioned on the previous page, i.e., a trip from London to Manchester, UK (200 miles, 4 h 40 min estimated travel time): What price would represent a good value (is appropriate) for you? The average price for a similar distance on this platform is 22 GBP. (open field)
What price would be expensive, yet still acceptable? (open field)
What price would be too cheap, thus raising doubts about quality? (open field)
What price would be too expensive, thus ruling out any consideration of booking? (open field)
**Importance of information (attributes)** How important is the following information to be available on the platform to you when selecting a ridesharing offer? (5-point Likert scale; 1 = not important at all; 2 = of little importance; 3 = of average importance; 4 = very important; 5 = absolutely essential) Driver’s name, Driver’s photo, Driver’s verification of email and phone number, Driver’s driving experience, Driver’s preferences (music, smoker/non-smoker, level of sociability), Car’s model and color, Car’s photo, Car’s year of production, Co-travellers’ name, Co-travellers’ photo, Co-travellers’ preferences (music, smoker/non-smoker, level of sociability), Other (please specify)
**Name sorting** What origin and gender does the owner of the name most probably belong to? (1 = European Male; 2 = European Female; 3 = European Gender-neutral; 4 = Muslim Male; 5 = Muslim Female; 6 = Muslim Gender-neutral; 7 = None of the offered groups) William, Narges, Noah, Tima, Hady, Oscar, Harry, Ben, Eylül, Samira, Sahar, Ranim, Louis, Hayyan, Raphaël, Maya, Nathan, Gabriel, Isabella, Hala, Eymen, Mila, Elif, Ella, Soraya, Joram, Miray, Shayan, Yusuf, Najib, Poone, Shahram, Alice, Rifat, Hugo, Suske, Oliver, Miraç, Meryem, Ava, Nizar, Paul, Alan Poppy, Aditya, Elias, Lily, Defne, Rose, Nima, Charlie, Ali, Jade, Ahmet, Mohammad, Sydu, Rashid, Manon, Layla, Jules, Sofia, Lea, Olivia, Isla, Mehdi, Manal, Ömer, Mustafa, Mila, Lina, Maen, Reza, Cesar, Samir, Aya, Ewa, Arthur, Yaser, Emma, Jack, Aischa, Uri, Ebrar, Asel, Marie, Anna, Hila, Zahra, Amelia, Hanna, Louise, Henry, Aseel, Amena, Jonas, Faezeh, Fawad, Qazal, Quynh, Emilia, Felix, Finn, Najme, George, Adam, Jacob, Lucas, Mia, Hiranur, Emir, Zeynep, Chloé, Leo, Fazal, Raheleh, Usama, Yiğit

5 and

**Table 6:** Results of the pre-test

Top typical European descent male names		Top typical European descent female names		Top typical Middle Eastern descent male names		Top typical Middle Eastern descent female names	
Name	Percentage of assignments into group (%)	Name	Percentage of assignments into group (%)	Name	Percentage of assignments into group (%)	Name	Percentage of assignments into group (%)
Adam^a^	94	Emma	92	Mohammad	96	Samira	68
Arthur	92	Lily	90	Yusuf	86	Aditya	68
Henry	92	Anna	86	Ahmet	84	Nima	66
Oliver	90	Olivia	86	Mustafa	84	Aischa	64
Jacob	90	Isabella	8	Samir	80	Zahra	60
William	88	Emilia	84	Emir	78	Soraya	60
Alan	88	Sofia	84	Rashid	78	Hala	58
Jack	88	Chloé	84	Yaser	74	Isla^b^	54
George	88	Alice	82	Ömer	72	Amena	50
Ben	86	Rose	82	Fawad	72		
Paul	86	Marie	82				
Felix	86	Hanna	80				
Lucas	86	Amelia	80				

^a^Excluded from further analysis for ambiguity reason: the name is perceived in Europe as a biblical name and in Middle East as Arabic phrase "made from."

^b^Was originally returned as European descent name

6.

## Appendix B: Overview of study procedures

See Fig. 7

## Appendix C: Descriptive statistics of the sample

See Table

**Table 7:** Sample characteristics in terms of age, income, and gender

Age group					
18–19 years	20–29 years	30–39 years	40–49 years	50–59 years	60–69 years
16 (6.0%)	90 (34.0%)	74 (27.9%)	49 (18.5%)	25 (9.4%)	11 (4.2%)
Income group (yearly net income)					
Less than £20,000	£20,000 to £34,999	£35,000 to £49,999	£50,000 to £74,999	£75,000 to £99,999	Over £100,000
129 (48.7%)	89 (33.6%)	27 (10.2%)	13 (4.9%)	6 (2.3%)	1 (0.4%)
Gender					
Female		Male		Other	
154 (58.1%)		108 (40.8%)		3 (1.1%)	

7.

## Appendix D: Post-hoc test for names grouping

We conducted a post-hoc test to double-check the perceptions of names descent. We asked 101 independent raters from Europe on the Prolific platform (www.prolific.co, 2021) to sort the names into categories: “Names can be of a different origin (descent). Moreover, there are typical male, female, and gender-neutral names. To what group do the following names most probably belong with regard to origin and gender?” (1 = European descent male; 2 = European descent female; 3 = European descent gender-neutral; 4 = Middle Eastern descent male; 5 = Middle Eastern descent female; 6 = Middle Eastern descent gender-neutral 7 = None of the offered groups). The results overwhelmingly confirm the grouping and are listed in the following (Table

**Table 8:** Results of post-hoc name sorting

Name	Group							Most likely group in post-hoc	Most likely group in pre-test and experiment
	E_Male (%)	E_Female (%)	E_GN (%)	ME_Male (%)	ME_Female (%)	ME_GN (%)	None (%)		
Jacob	**66**	0	1	31	0	1	1	E_Male	E_Male
Alan	**93**	1	3	2	0	0	1	E_Male	E_Male
Samira	1	3	0	5	**85**	4	2	ME_Female	ME_Female
Amelia		**77**	1	0	16	0	1	E_Female	E_Female
George	**95**	0	2	3	0	0	0	E_Male	E_Male
Emilia	3	**76**	1	0	16	2	2	E_Female	E_Female
Mustafa	0	0	0	**92**	2	2	4	ME_Male	ME_Male
Rashid		0	0	**95**	1	3	1	ME_Male	ME_Male
Isabella	10	**82**	1	0	7	0	0	E_Female	E_Female
Ahmet	0	0	1	**92**	2	5	0	ME_Male	ME_Male
Zahra	0	6	1	6	**76**	9	2	ME_Female	ME_Female
Oliver	**96**	0	2	2	0	0	0	E_Male	E_Male
Hanna	3	**71**	0	0	24	0	2	E_Female	E_Female
Soraya	1	2	1	3	**81**	7	5	ME_Female	ME_Female
Emir	2	0	0	**85**	4	7	2	ME_Male	ME_Male
Henry	**97**	2	0	1	0	0	0	E_Male	E_Male
Felix	**92**	0	3	0	0	0	5	E_Male	E_Male
Mohammad	0	0	0	**98**	1	0	1	ME_Male	ME_Male
William	**98**	0	0	1	0	0	1	E_Male	E_Male
Ben	**90**	0	1	8	0	1	0	E_Male	E_Male
Olivia	2	**94**	2	0	2	0	0	E_Female	E_Female
Aischa	0	6	1	0	**78**	6	9	ME_Female	ME_Female
Chloé	7	**86**	0	0	4	0	3	E_Female	E_Female
Yaser	0	0	0	**67**	6	26	1	ME_Male	ME_Male
Isla	1	**49**	2	3	**31**	7	8	E_Female	ME_Female
Jack	**95**	0	0	4	0	0	1	E_Male	E_Male
Samir	1	0	0	**83**	8	8	0	ME_Male	ME_Male
Emma	5	**91**	0	0	3	0	1	E_Female	E_Female
Sofia	1	**73**	0	1	24	0	1	E_Female	E_Female
Rose	4	**92**	2	0	2	0	0	E_Female	E_Female
Marie	4	**90**	0	0	6	0	0	E_Female	E_Female
Ömer	3	0	1	**88**	0	6	2	ME_Male	ME_Male
Amena	0	2	0	1	**88**	5	4	ME_Female	ME_Female
Arthur	**98**	1	0	0	0	1	0	E_Male	E_Male
Aditya	0	1	0	9	**61**	18	11	ME_Female	ME_Female
Yusuf	0	0	0	**94**	1	3	2	ME_Male	ME_Male
Hala	0	2	0	11	**48**	29	11	ME_Female	ME_Female
Paul	**96**	0	0	3	1	0	0	E_Male	E_Male
Alice	3	**93**	2	0	0	1	1	E_Female	E_Female

*E_Male* European descent male name, *E_Female* European descent female name, *E_GN* European descent gender-neutral name, *ME_Male* Middle Eastern descent male name, *ME_Female* Middle Eastern descent female name, *ME_GN* Middle Eastern descent gender-neutral name

8).
